# Interferon Alpha Induces Cellular Autophagy and Modulates Hepatitis B Virus Replication

**DOI:** 10.3389/fcimb.2022.804011

**Published:** 2022-02-02

**Authors:** Jia Li, Thekla Kemper, Ruth Broering, Jieliang Chen, Zhenghong Yuan, Xueyu Wang, Mengji Lu

**Affiliations:** ^1^ Insititute for Virology, University Hospital Essen, University of Duisburg-Essen, Essen, Germany; ^2^ Department of Gastroenterology, Hepatology and Transplant Medicine, University Hospital Essen, University of Duisburg-Essen, Essen, Germany; ^3^ Key Laboratory of Medical Molecular Virology, School of Basic Medical Sciences, Shanghai Medical College, Fudan University, Shanghai, China; ^4^ State Key Laboratory for Diagnostic and Treatment of Infectious Diseases, The First Affiliated Hospital, School of Medicine, Zhejiang University, Hangzhou, China

**Keywords:** Hepatitis B virus, IFNα-2a, Akt/mTOR signaling, AMPK, autophagy

## Abstract

Hepatitis B virus (HBV) infection causes acute and chronic liver diseases, including severe hepatitis, liver cirrhosis, and hepatocellular carcinoma (HCC). Interferon alpha 2a (IFNα-2a) is commonly used for treating chronic HBV infection. However, its efficacy remains relatively low. Yet, the immunological and molecular mechanisms for successful IFNα-2a treatment remain elusive. One issue is whether the application of increasing IFNα doses may modulate cellular processes and HBV replication in hepatic cells. In the present study, we focused on the interaction of IFNα signaling with other cellular signaling pathways and the consequence for HBV replication. The results showed that with the concentration of 6000 U/ml IFNα-2a treatment downregulated the activity of not only the Akt/mTOR signaling but also the AMPK signaling. Additionally, IFNα-2a treatment increased the formation of the autophagosomes by blocking autophagic degradation. Furthermore, IFNα-2a treatment inhibited the Akt/mTOR signaling and initiated autophagy under low and high glucose concentrations. In reverse, inhibition of autophagy using 3-methyladenine (3-MA) and glucose concentrations influenced the expression of IFNα-2a-induced ISG15 and IFITM1. Despite of ISGs induction, HBV replication and gene expression in HepG2.2.15 cells, a cell model with continuous HBV replication, were slightly increased at high doses of IFNα-2a. In conclusion, our study indicates that IFNα-2a treatment may interfere with multiple intracellular signaling pathways, facilitate autophagy initiation, and block autophagic degradation, thereby resulting in slightly enhanced HBV replication.

## Introduction

Hepatitis B virus (HBV) infection causes acute and chronic infections that results in the death of over 887,000 people every year due to severe hepatitis, liver cirrhosis, and cancer despite of effective vaccination (Polaris Observatory, 2018). Current standard HBV therapeutic strategies include treatment using interferon alpha (IFNα) (Ferir et al., 2008) and nucleoside/nucleotide analogs (NAs) (Terrault et al., 2018). IFNα-2a is a cytokine with immunomodulatory and antiviral effects and is one of the first-line drugs for clinical treatment of chronic hepatitis B (CHB) (Cho and Kelsall, 2014). Nevertheless, the performance of IFNα-2a-based therapies has been limited, with a response rate of only approximately 30% (Ferir et al., 2008). The mechanisms underlying the limited response to IFNα in CHB patients are not completely understood.

The major human type I IFN includes three subtypes: α, β, and ω, primarily exerting antiviral, antiproliferative, and immunomodulatory functions (Wittling et al., 2020). IFNα binds to its specific receptors and activates intracellular signaling cascades (Pestka et al., 2004). A great number of cellular genes are up-or downregulated by IFNα and many of those so-called IFN-stimulated genes (ISGs) have antiviral functions (Schoggins and Rice, 2011; Schneider et al., 2014). The antiviral effect of ISGs in the context of HBV infection has been extensively studied over decades (Wieland et al., 2003). Overexpression of selected ISGs may lead to HBV suppression in cell models (Pei et al., 2014; Leong et al., 2016). Though many ISGs may contribute to suppression of HBV replication, their actions were rather ineffective (Rang et al., 2001; Kim et al., 2008). For example, IFIT1 and -2 are the most strongly upregulated genes, however, they rather play a role in limiting HBV replication at a high level (Pei et al., 2014). IFNα subtypes differ in their antiviral and immunomodulatory functions (Chen et al., 2021). IFNα-14 has been shown to be superior in HBV inhibition compared to other IFNα subtypes including IFNα-2a (Shen et al., 2018; Chen et al., 2021). Mechanistically, IFNα-14 is able to trigger a concerted IFNα and –γ response and presumably a broader antiviral activity in hepatocytes. Besides the JAK/STAT, which are called canonical signaling pathways (Mazewski et al., 2020), IFNα is able to modulate multiple intracellular signaling pathways through non-canonical, including MAP kinase and phosphoinositide 3-kinase (PI3K)/mammalian target of rapamycin (mTOR) pathways (Mazewski et al., 2020). Specifically, IFNα activates PI3K/Akt/mTOR signaling pathway (Uddin et al., 1995; Lekmine et al., 2004) and therefore initiates cellular autophagy.

Autophagy is an evolutionarily conserved cellular process that can be induced by various stimuli, including starvation, hypoxia, and treatment with cytokines (such as IFN and transforming growth factor (TGF)) (Lekmine et al., 2004; Schmid and Munz, 2007; Chang et al., 2010). Autophagy is initiated by mTOR or AMPK-ULK1 signaling to regulate ULK1-Atg13-FIP200 complex formation (Jung et al., 2009). Recent evidence suggests that type I IFN can induce autophagy by canonical and non-canonical pathways and participate in many pathological processes (Degtyarev et al., 2008; Zhu et al., 2013; Gu et al., 2017; Perot et al., 2018; Ma et al., 2019), which is correlated with cellular apoptosis and proliferation (Degtyarev et al., 2008; Zhu et al., 2013), cytokines networks (Gu et al., 2017; Ma et al., 2019), and antiviral activities (Perot et al., 2018).

Our and others’ previous studies showed that autophagy plays a key role in HBV replication and pathogenesis (Tian et al., 2011; Lin et al., 2017). Therefore, it is important to investigate whether the intracellular crosstalk induced by IFNα-2a can enhance cellular autophagy and change HBV replication in hepatic cells. In the present study, we tested the changes in intracellular signaling pathways and HBV replication after IFNα-2a treatment in hepatoma cells, HepG2.2.15 and Huh7 cells, which are commonly used cell models for HBV replication, and primary human hepatocytes (PHHs). The results illustrate that IFNα-2a induced autophagy and blocked autophagic degradation, resulting in a slight increase in HBV replication.

## Materials and Methods

### Cell Culture

All the cell cultures were maintained at 37°C in a humidified atmosphere containing 5% CO2. The HBV-producing HepG2.2.15 hepatoma cell line, which harbors the integrated HBV genomic dimers (Sells et al., 1987) (provided by American Type Culture Collection), was cultured in RPMI-1640 medium (Gibco) with 10% fetal bovine serum, 100 U/ml penicillin, 100 μg/ml streptomycin (Gibco), and 1% nonessential amino acids (NEAAs), 1% HEPES, and 500 μg/ml G418 (Merck Millipore). Different concentrations of glucose were used in HepG2.2.15 culture medium, based on glucose‐free RPMI‐1640 medium (11879020; Gibco), supplemented with the indicated concentrations of glucose (5 and 25 mM), 10% fetal bovine serum, 100 U/ml penicillin, 100 μg/ml streptomycin (Gibco), 1% NEAAs, 1% HEPES, and 500 μg/ml G418 (Merck Millipore). Huh7 cells were cultured in DMEM medium, supplemented with 10% inactivated FBS, 100 U/mL penicillin, 100 μg/mL streptomycin (Gibco), 1% NEAA, and 1% HEPES. Primary human hepatocytes (PHHs) were cultured in Williams E medium, supplemented with 250 μL insulin, 2% DMSO, and 125 μL hydrocortisone hemisuccinate. The HBV particles harvested from HepG2.117 cells were used for PHHs infection. For HBV infection, as described previously (Wan et al., 2017). PHHs were cultured in primary hepatocyte maintenance medium (PMM) for 24 hours and then inoculated with a 30 multiplicity of genome equivalents of HBV in PMM with 4% PEG 8000 at 37°C for about 24 hours. One day after infection, the cells were washed with warm PBS three times to remove residual viral particles and refreshed the PMM medium containing 2% DMSO and treated with the indicated concentrations of IFNα-2a. The medium was refreshed every other day.

### Chemical Reagents

Human interferon alpha 2a (11100) was purchased from PBL assay science. Glucose (G8270), chloroquine (C6628), insulin (I-034), and rapamycin (R8781) were purchased from Sigma‐Aldrich. MHY1485 (S7811) and AICAR (S1802) were purchased from Selleck Chemicals.

### Western Blotting Assay

Western blotting assay was carried out as described previously (Lin et al., 2020). Antibodies we used in the experiment were as following: anti-AMPK (2532S), anti-phospho-AMPK (2531S), anti-Akt (9272S), anti-phospho-Akt (Ser473; 9271S), anti-mTOR (2972S), anti-phospho-mTOR (2971S), anti-p70 S6K (9202S), anti-phosphop70 S6K (S371; 9234S), anti-p62 (5114S), anti-LC3B (3868S), anti-IFITM1 (13126S), anti-ISG15 (2758S) and anti-beta-actin (Sigma, A5441). The signals were visualized with an enhanced chemiluminescence (ECL) system (GE Healthcare, UK). The relative levels of indicated proteins were determined by quantifying the gray scales of bands using ImageJ software, using beta‐actin as a loading control.

### Immunofluorescence Staining

HepG2.2.15 cells were grown on coverslips and treated as indicated in each experiment. After treatment, the cells were fixed in 4% paraformaldehyde and permeabilized with 0.1% Triton X‐100. Then, the cells were incubated with anti-LC3B antibodies, staining with Alexa Fluor 488‐conjugated Goat anti-Rabbit IgG (H + L). The nuclei were stained with 4’,6-Diamidino-2-phenylindole (DAPI). The distribution of LC3B protein was visualized with a confocal microscope (LSM710, Carl Zeiss). For quantification the number of LC3B puncta in cells, around 3 cells were recorded and analyzed by ImageJ software.

### Dye Quenched-Bovine Serum Albumin (DQ-BSA) Degradation Assay

HepG2.2.15 cells were grown on coverslips and treated as indicated in each experiment. After treatment, cells were incubated with DQ Red BSA (10 μg/mL; Invitrogen, D-12051) for 30 minutes. The fluorescent signal produced by lysosomal proteolysis of DQ Red BSA was quantified with an LSM 710 confocal microscope (Zeiss, Germany).

### Detection of HBV Gene Expression and Replication

The levels of HBsAg and HBeAg in cell lysates and in the supernatant were measured by chemiluminescent microparticle immunoassay (CMIA, Abbott Laboratories, Chicago, IL, USA). HBV replicative intermediates (RIs) from intracellular core particles were extracted and detected by Southern blotting as described previously (Guan et al., 2007).

### Statistical Analyses

All experiments were repeated independently at least 3 times. The data were expressed as the means ± SEM. Data were analyzed for statistical significance by ANOVA with two‐tailed Student’s t test or by one‐way ANOVA with a Tukey post-test using Prism 8 software. p < 0.05 was considered significant and indicated by asterisks.

## Results

### IFNα-2a Interferes Intracellular Signal Crosstalk

**Figure 1:**
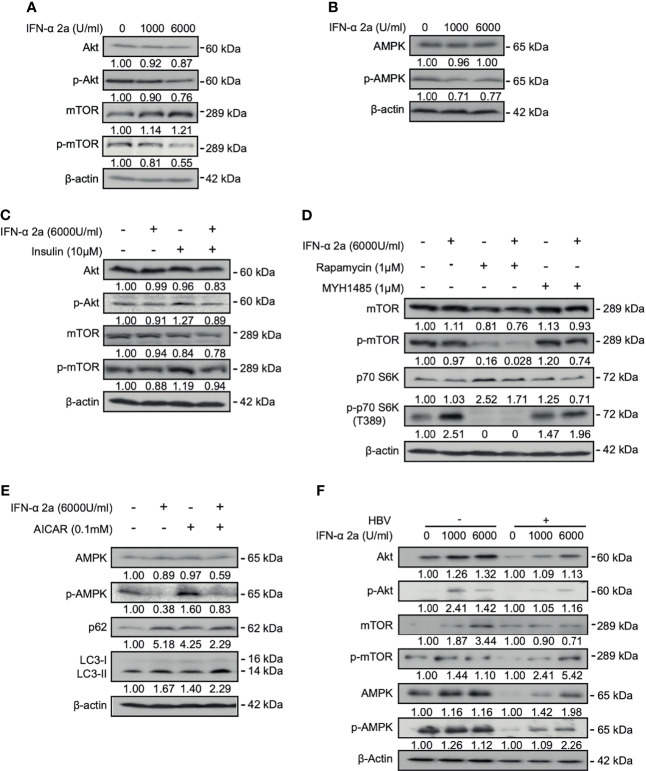
IFNα-2a interferes with intracellular signal crosstalk. **(A, B)** HepG2.2.15 cells were treated with the indicated concentrations of IFNα-2a and harvested after 48 h. The total and phosphorylated Akt, mTOR, and AMPK were detected by western blotting and their relative levels were determined by quantifying the gray scales of bands, using beta‐actin as a loading control. **(C)** HepG2.2.15 cells were treated with 6000 U/ml IFNα-2a in combination with 10 μM insulin for 48 hr. The total and phosphorylated Akt, and mTOR were detected by western blotting and their relative levels were determined by quantifying the gray scales of bands, using beta‐actin as a loading control. **(D)** HepG2.2.15 cells were treated with 6000 U/ml IFNα-2a with or without 1 μM MTOR inhibitor rapamycin or activator MHY1485 for 48 h The total and phosphorylated mTOR and p-p70 S6K were detected by western blotting and their relative levels were determined by quantifying the gray scales of bands, using beta‐actin as a loading control. **(E)** HepG2.2.15 cells were treated with 6000 U/ml IFNα-2a with or without AMPK activator 0.1 mM AICAR for 48 hr. AMPK, phosphorylated AMPK, p62 and LC3 were analyzed by western blotting, using beta‐actin as a loading control. **(F)** PHHs were infected with HBV virions (multiplicity of infection = 30). 4 days post infection, PHHs were treated with IFNα-2a twice (1000 U/ml and 6000 U/ml). After 48 hr, The total and phosphorylated Akt, mTOR, and AMPK were detected by western blotting and their relative levels were determined by quantifying the gray scales of bands, using beta‐actin as a loading control.

Previously, it has been shown that IFNα-2a was able to interfere with multiple cellular signalings. We firstly asked whether IFNα-2a interferes with Akt/mTOR and AMPK activities. HepG2.2.15 cells were treated with IFNα-2a at different concentrations (1000 U/ml and 6000 U/ml), and harvested after 48 hr. The baseline expressions of total AMPK, Akt, mTOR, and their phosphorylated forms were detected by western blotting. As the **Figure 1A** shows, the levels of phosphorylated Akt and mTOR were decreased with the increasing IFNα-2a concentrations. Interestingly, the levels of total and phosphorylated AMPK were decreased (**Figure 1B**). These data indicate that IFNα-2a attenuates Akt/mTOR signaling and AMPK signaling.

Previous studies have shown that insulin physiologically activates the Akt/mTOR pathway (Molinaro et al., 2019). Insulin was added into the culture medium. Indeed, the levels of phosphorylated Akt and mTOR were increased after insulin treatment. Simultaneous addition of 6000 U/ml IFNα-2a counteracted the increased activities of Akt and mTOR stimulated by insulin (**Figure 1C**), confirming the suppressive effect of IFNα-2a on Akt/mTOR signaling.

To further examine the effect of IFNα-2a on mTOR signaling pathway, HepG2.2.15 cells were treated with 6000 U/ml of IFNα-2a combined either with rapamycin, an inhibitor of mTOR, or MHY1485, an agonist of mTOR, respectively. The results showed that the level of phosphorylated mTOR decreased after rapamycin treatment and was further reduced following the combined treatment with IFNα-2a and rapamycin. On the other hand, the inhibitory effect of IFNα-2a on mTOR phosphorylation was attenuated in cells co-treated with MHY1485 (**Figure 1D**). These results confirm that IFNα-2a treatment inhibits the mTOR signaling pathway in hepatoma cells.

IFNα-2a treatment resulted in a significant decrease of AMPK phosphorylation at the baseline expression. When HepG2.2.15 cells were grown in the presence of AICAR, an AMPK agonist, for 48 hr, AMPK phosphorylation markedly increased. Again, IFNα-2a co-treatment abolished increased phosphorylation of AMPK by AICAR activation (**Figure 1E**). Thus, IFNα-2a treatment also diminishes AMPK signaling in hepatoma cells.

Besides the hepatoma cells, we also used PHHs in our experiment. However, uninfected PHHs had initially very low levels of phosphorylated Akt and mTOR. PHHs were incubated in medium and treated with indicated concentrations of IFNα-2a (1000 U/ml and 6000 U/ml) for 48 hr. Differed from HepG2.2.15 cells, IFNα-2a treatment caused increased levels of phosphorylated Akt, mTOR, and AMPK in PHHs (**Figure 1F**). HBV-infected PHHs cell model is a transient and *de novo* infection model and is notably different from the stable HBV-transfected cells, such as HepG2.2.15 cells.

### IFNα-2a Induces Autophagy Through Inhibiting Akt/mTOR Pathway and Blocks Autophagic Degradation in Hepatoma Cells

**Figure 2:**
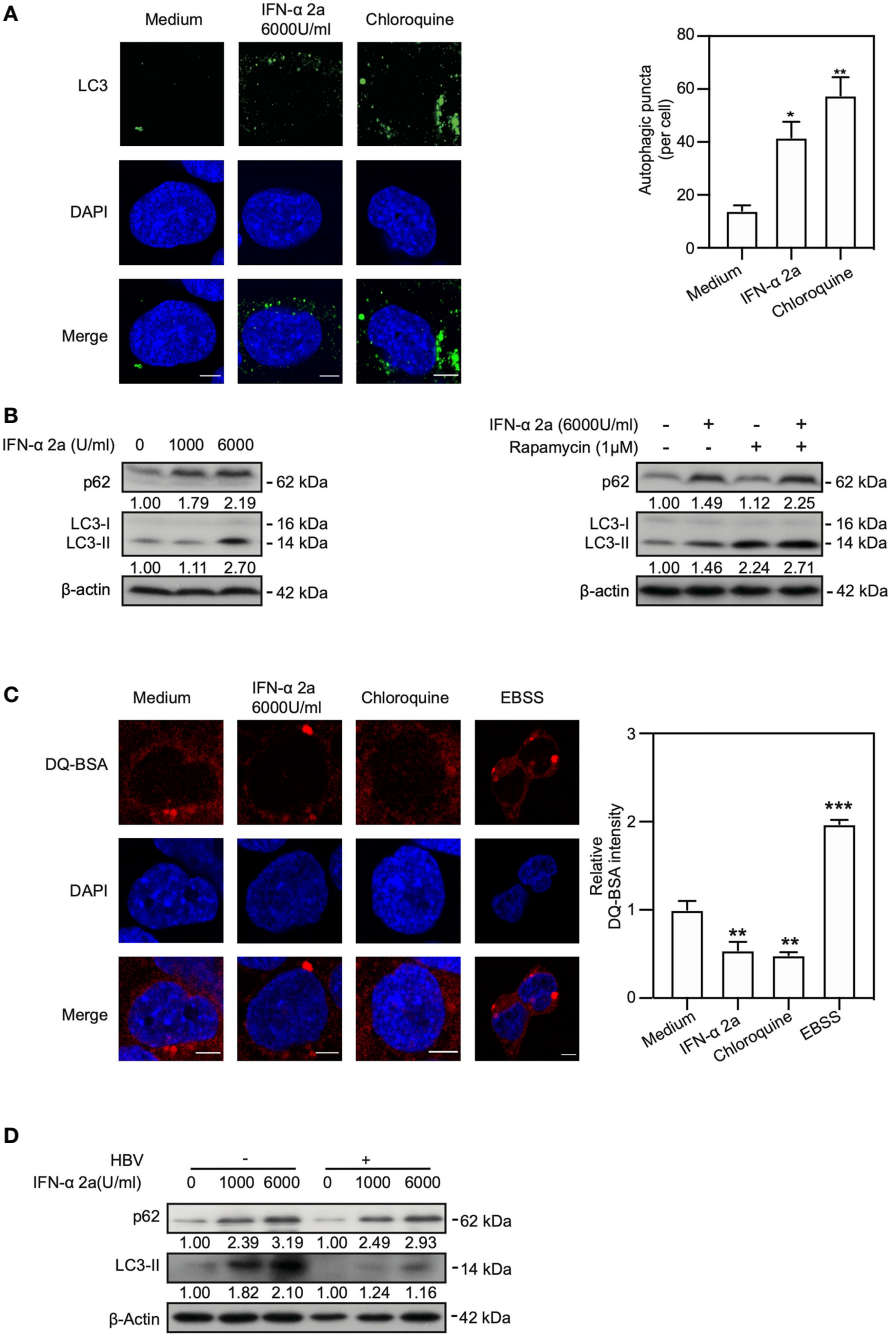
IFNα-2a induces autophagy through inhibiting Akt/mTOR pathway and blocks autophagic degradation. **(A)** HepG2.2.15 cells were treated with 6000 U/ml IFNα-2a. After 48 hr, cells were fixed, incubated with rabbit anti-LC3 antibodies, followed by staining with Alexa Fluor 488-conjugated anti-rabbit secondary antibody IgG. Finally, the distribution of LC3 was imaged by confocal microscopy. Cells treated with 10 nM chloroquine (CQ) were used as a positive control. LC3 puncta in cells were quantified as described previously (Lin et al., 2020). Scale bar: 5 μm. **(B)** HepG2.2.15 cells were treated with the indicated concentrations of IFNα-2a and harvested after 48 hr. HepG2.2.15 cells were cultured in the medium either with 6000 U/ml IFNα-2a or rapamycin (1 μM) for 48 hr. p62 and LC3 were analyzed by western blotting using beta‐actin as a loading control. The relative levels were determined by quantifying the gray scales of bands, using beta‐actin as a loading control. **(C)** HepG2.2.15 cells were treated with 6000 U/ml IFNα-2a for 48 hr, followed by incubation with 10 μg/mL DQ-BSA for 30 minutes. The fluorescent signal of DQ-BSA was detected by confocal microscopy. Cells cultured with EBSS for 2 hours were used as a positive control and CQ treatment was as a negative control. Scale bar: 5 μm. **(D)** PHHs were infected with HBV virions (multiplicity of infection = 30). 4 days post infection, PHHs were treated with IFNα-2a twice (1000 U/ml and 6000 U/ml). After 48 hr, p62 and LC3 were analyzed by western blotting using beta‐actin as a loading control. *P < 0.05; **P < 0.01; ***P < 0.001.

The Akt/mTOR signaling pathway is known to regulate autophagy (Degtyarev et al., 2008; Jung et al., 2009). Thus, we addressed the question whether IFNα-2a treatment changes the autophagic activity in hepatoma cells. The levels of LC3II after IFNα-2a treatment were determined using IF staining and western blotting. IF staining showed that the numbers of endogenous LC3-positive autophagic puncta increased in IFNα-2a-treated cells (**Figure 2A**). Consistently, the IFNα-2a treatment also markedly increased the levels of LC3II, as shown by western blotting analysis (**Figure 2B**). Next, HepG2.2.15 cells were cultured in the presence of rapamycin with and without IFNα-2a, respectively. The level of LC3II was higher after rapamycin treatment and further increased by IFNα-2a co-treatment. Interestingly, the level of p62 also increased markedly after IFNα-2a treatment, similar to chloroquine (CQ) treatment. These results indicate that IFNα-2a treatment blocked autophagic degradation and led to accumulation of LC3 puncta. To confirm this assumption, HepG2.2.15 cells were treated with 6000 U/ml IFNα-2a for 48 hr and then incubated with dye quenched-bovine serum albumin (DQ-BSA) for 30 min. Cells cultured in Earle’s balanced salt solution (EBSS) was used as a positive control and CQ treatment as a negative control. Fluorescence microscopic analysis showed that the fluorescent signal of DQ-BSA decreased upon IFNα-2a treatment, while increased upon incubation with Earle’s balanced salt solution (EBSS), suggesting that IFNα-2a treatment blocks autophagic degradation in hepatoma cells (**Figure 2C**).

Next, we explored whether IFNα-2a treatment induce autophagy in PHHs. Interestingly, consistent with the previous results, the levels of p62 and LC3 were upregulated after being posed to IFNα-2a, indicating an increased level of autophagy and a decreased autophagic degradation in PHHs (**Figure 2D**).

Taken together, these results demonstrate that IFNα-2a treatment promotes the formation of autophagosomes and blocks the autophagic degradation in lysosomes.

### IFNα-2a Inhibits Akt/mTOR Activation and Enhances Autophagy Independently on Glucose Concentrations

**Figure 3:**
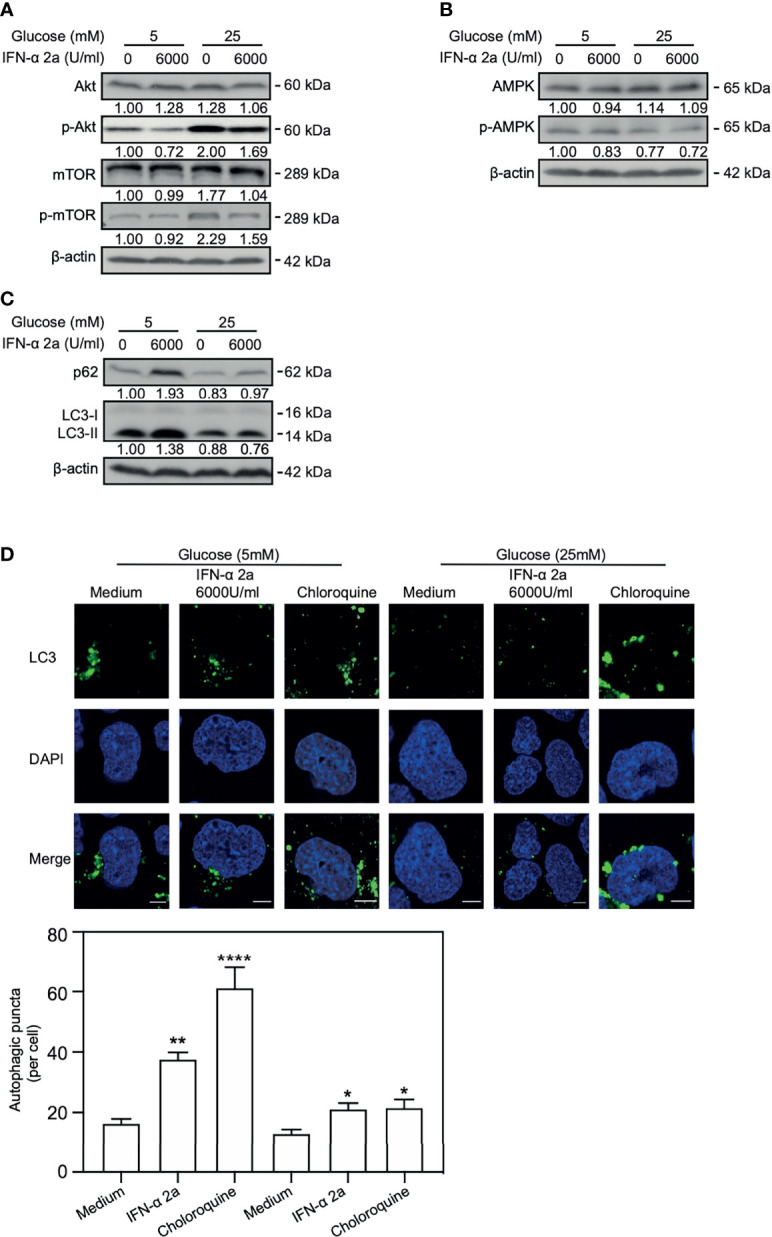
IFNα-2a inhibits Akt/mTOR activation and enhances autophagy independently on glucose concentrations. HepG2.2.15 cells were cultured in medium with the indicated glucose concentrations (5 and 25 mM) with or without 6000U/ml IFNα-2a and harvested after 48 hr. **(A)** The total and phosphorylated Akt, mTOR, **(B)** AMPK and **(C)** p62 and LC3 were detected by western blotting and their relative levels were determined by quantifying the gray scales of bands, using beta‐actin as a loading control. **(D)** The cells were fixed, incubated with rabbit anti-LC3 antibodies, followed by staining with Alexa Fluor 488-conjugated anti-rabbit secondary antibody IgG. Finally, the distribution of LC3 was imaged by confocal microscopy. The results presented in the graphs were calculated from at least five cells. Scale bar: 5 μm. *P < 0.05; **P < 0.01; ****P < 0.001.

Cellular energy metabolism can modulate cellular autophagy (Meijer and Codogno, 2011). Our previous study showed that high glucose concentration reduced autophagy by activating Akt/mTOR pathway. On the contrary, autophagy is enhanced by activation of AMPK and ULK1 and inhibition of Akt, mTOR and p70 S6K at low glucose concentration (Wang et al., 2020). Therefore, we asked whether glucose concentrations also influence IFNα-2a-mediated Akt/mTOR inhibition and enhancement of autophagy. HepG2.2.15 cells were cultured with the indicated glucose concentrations (5 mM and 25 mM, later called low glucose and high glucose conditions, respectively) and treated with 6000 U/ml IFNα-2a for 48 hr. Western blotting was used to detect the levels of total Akt, mTOR, and AMPK proteins and their phosphorylated forms. Consistent with our previous results, the levels of the phosphorylated Akt and mTOR were increased under the high glucose condition (**Figures 3A** and **S1**) but decreased in hepatoma cells in the presence of IFNα-2a in hepatoma cells. In addition, AMPK was inactivated under the high glucose condition and much less active when additionally treated with IFNα-2a (**Figure 3B**). These data suggest that IFNα-2a-mediated Akt/mTOR inhibition occurred independently on glucose concentrations.

Consistently, independent of the glucose concentrations, the levels of LC3II and p62 and the numbers of LC3-positive autophagic puncta increase in IFNα-2a-treated HepG2.2.15 cells (**Figures 3C, D**) and Huh7 cells (**Figure S1**).

Taken together, IFNα-2a inhibits Akt/mTOR activation and enhances autophagy independently on glucose concentrations in hepatoma cells.

### IFNα-2a Induced ISGs Expression Is Dependent on Autophagy and Glucose

**Figure 4:**
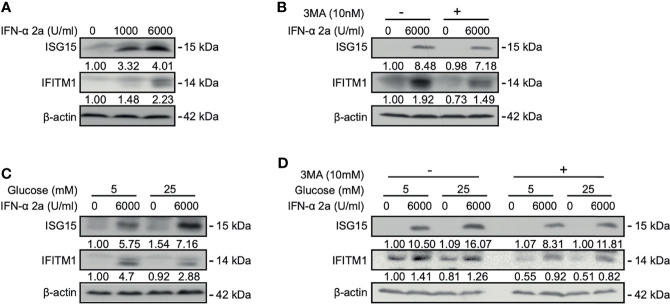
IFNα-2a induced ISGs expression is dependent on autophagy and glucose concentrations. **(A)** HepG2.2.15 cells were treated with the indicated concentrations of IFNα-2a and harvested after 48 hr. **(B)** HepG2.2.15 cells were treated with 6000 U/ml IFNα-2a with or without 10 nM 3-MA and harvested after 48 hr. **(C)** HepG2.2.15 cells were cultured in medium with the indicated glucose concentrations (5 and 25 mM) with or without 6000 U/ml IFNα-2a and harvested after 48 hr. **(D)** HepG2.2.15 cells were cultured at the indicated glucose concentrations and 6000 U/ml IFNα-2a with or without 10 nM 3-MA. The levels of ISG15 and IFITM1 were detected by western blotting, and their relative levels were determined by quantifying the gray scales of bands, using beta‐actin as a loading control.

Interferon-stimulated 15 kDa protein (ISG15) and interferon-induced transmembrane protein1 (IFITM1) are the typical anti-viral proteins under the control of IFN signaling and highly expressed after IFN stimulation. IFN-α treatment activates the JAK–STAT pathway and upregulates the expression of ISGs (Darnell et al., 1994; van Boxel-Dezaire et al., 2006). Activation of the JAK-STAT pathway could be verified by the detection of increased levels of STAT1 and phosphorylated STAT1 in our cell model (**Figure S2**). Further, emerged evidence implicated that PI3K/Akt/mTOR pathway is also involved in the induction of ISGs (Kaur et al., 2008; Kaur et al., 2012). We asked whether PI3K/Akt/mTOR signal pathways-related autophagy may also participate in the regulation of ISG15 and IFITM1 expression. Western blotting analysis showed that the expressions of ISG15 and IFITM1 were upregulated after IFNα-2a treatment (**Figure 4A**). When using an autophagy inhibitor 3-Methyladenine (3-MA), ISG15 and IFITM1 were reduced (**Figure 4B**), suggesting a positive effect of autophagy on ISG15 and IFITM1 induction.

As glucose can modulate Akt/mTOR/ULK1 signaling pathway and regulate autophagy (Wang et al., 2020), we tested whether glucose-mediated autophagy also influences ISG15 and IFITM1 expression. HepG2.2.15 cells were cultured in medium with low and high glucose concentrations, respectively, and then treated with IFNα-2a. Interestingly, IFNα-2a differently promoted ISG15 and IFITM1 expression at high glucose concentration, if compared with their corresponding expression levels at the low glucose concentration (**Figure 4C**).

Moreover, HepG2.2.15 cells were treated with 3-MA at the indicated glucose concentrations, combined with IFNα-2a treatment. Consistently, both the expressions of ISG15 and IFITM1 were significantly suppressed by the co-treatment of 3-MA and IFNα-2a. It can be further verified ISG15 and IFITM1 expression were dependent on autophagy. These findings illustrate that IFNα-2a-induced autophagy and glucose concentrations influence ISGs expression, however, the accurate mechanisms need to be investigated in the future.

### High IFNα-2a Concentrations Do Not Inhibit HBV Replication and Gene Expression in Hepatoma Cells

**Figure 5:**
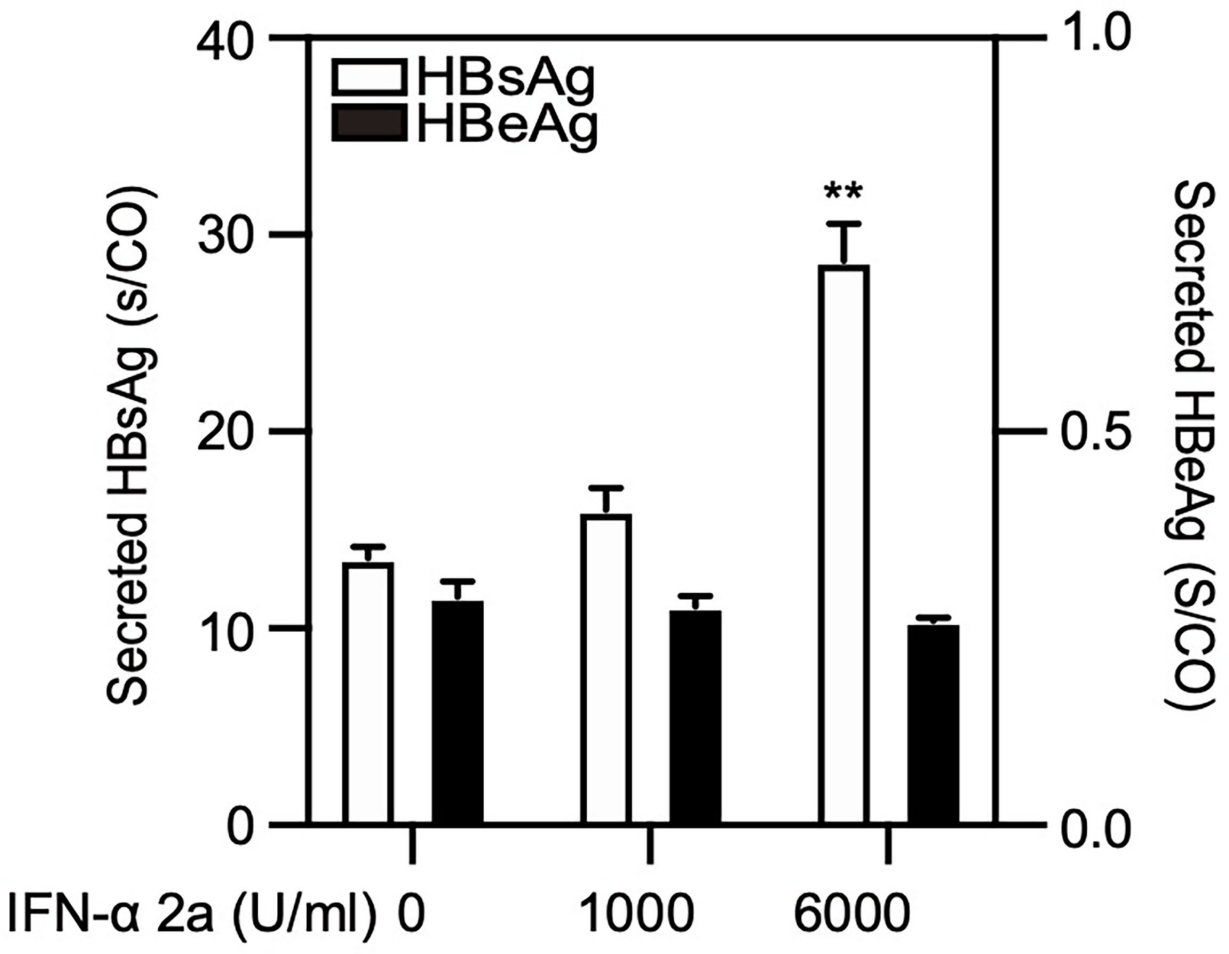
High IFNα-2a concentrations promote the yield of HBsAg in PHHs. PHHs were infected with HBV virions (multiplicity of infection = 30). 4 days post infection, PHHs were treated with IFNα-2a twice (1000 U/ml and 6000 U/ml). After 48 hr, the HBsAg and HBeAg levels in the culture supernatants were harvested and quantified by chemiluminescent microparticle immunoassay (CMIA). **P < 0.01.

Cellular autophagy is an important process for HBV life cycle by regulating HBV transcription, assembly, and release (Lin et al., 2017; Lin et al., 2020). Thus, we explored whether IFNα-2a-induced autophagy has an impact on HBV replication. PHHs were infected with HBV and treated with IFNα-2a post infection. The levels of HBsAg and HBeAg in the culture supernatants were detected by CIMA. After the treatment at the concentration of 6000 U/ml IFNα-2a, the HBsAg increased significantly (**Figure 5**). A short-term (24 h) IFNα-2a treatment of transiently pSM2-transfected Huh7 did not significantly change the HBsAg production, likely due to the short time period (**Figure S3A**).

**Figure 6:**
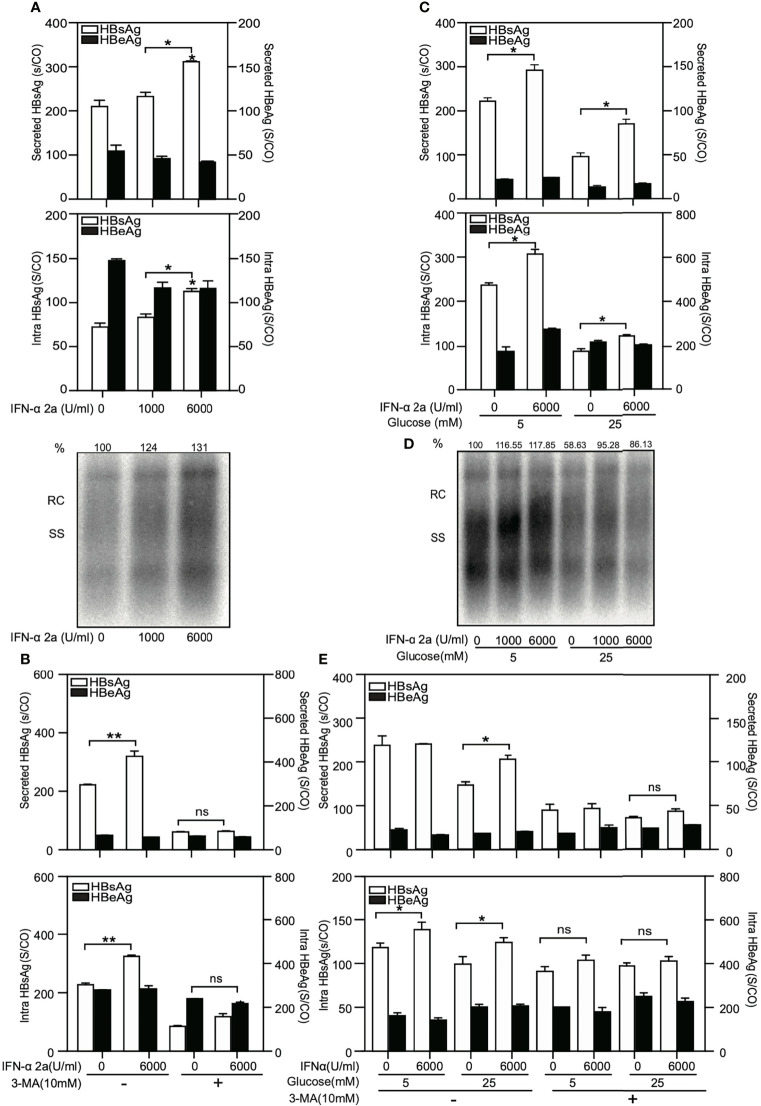
High IFNα-2a concentrations do not inhibit HBV replication and gene expression in HepG2.2.15 cells. **(A)** HepG2.2.15 cells were cultured in medium with the indicated concentrations of IFNα-2a. **(B)** HepG2.2.15 cells were treated with 6000 U/ml IFNα-2a with or without 10 nM 3-MA. **(C)** HepG2.2.15 cells were cultured in medium with the indicated glucose concentrations (5 and 25 mM) and treated with 6000 U/ml IFNα-2a. After 72 hr, cells were harvested and the HBsAg and HBeAg levels in the culture supernatants and intracellular HBsAg and HBeAg from cell lysates were quantified by chemiluminescent microparticle immunoassay Å(CMIA). **(D)** HepG2.2.15 cells were cultured at the indicated glucose concentrations (5 and 25 mM) and IFNα-2a (1000 U/ml and 6000 U/ml). Encapsidated HBV RIs were detected by Southern blotting. **(E)** HepG2.2.15 cells were cultured at the indicated glucose concentrations (5 and 25 mM) and 6000 U/ml IFNα-2a with or without 10 nM 3-MA. After 72 hr, cells were harvested and the HBsAg and HBeAg levels in the culture supernatants and intracellular HBsAg and HBeAg from cell lysates were quantified by chemiluminescent microparticle immunoassay (CMIA). *P < 0.05; **P < 0.01; ns, not significant; RC, relaxed circular DNA; S/CO, signal to cutoff ratio; SS, single‐stranded DNA.

HepG2.2.15 cells were treated with IFNα-2a at different concentrations (1000 U/ml and 6000 U/ml), and harvested after 72 hr. The levels of secreted and intracellular HBsAg and HBeAg were measured by chemiluminescent microparticle immunoassay. HBV replicative intermediates (RIs) were detected by Southern blotting. The levels of secreted and intracellular HBsAg but not HBeAg as well as the amount of HBV RIs increased after IFNα-2a treatment (**Figure 6A**).

These data suggest that IFNα-2a has a poor antiviral response in HepG2.2.15 cells and infected PHHs, and has a slight but measurable virus-promoting effect at high concentrations.

Then, to test whether IFNα-2a-mediated enhancement of HBV replication is related to autophagy, HepG2.2.15 cells were co-treated with 3-MA and IFNα-2a. 3-MA clearly blocked the positive effect of IFNα-2a on HBsAg production and secretion (**Figure 6B**).

Next, HepG2.2.15 cells were grown at the indicated glucose concentrations and treated with IFNα-2a. The results showed that the secreted and intracellular HBsAg increased after IFNα-2a treatment both at low and high glucose conditions (**Figure 6C**). The amount of HBV RIs decreased at the high glucose concentration, but it increased after IFNα-2a treatment independent on glucose concentrations (**Figure 6D**). Consistently, the secreted and intracellular HBsAg decreased after co-treatment with 3-MA (**Figure 6E**). All these results indicate that IFNα-2a enhances HBV replication at a high dose and this enhancement is dependent on autophagy.

## Discussion

In the present study, we demonstrated that IFNα-2a interferes with multiple intracellular signaling pathways, including inhibiting Akt/mTOR and AMPK signaling pathways, promoting the autophagosomes formation, and blocking autophagic degradation. This action of IFNα-2a resulted in enhanced HBV replication. Additionally, the induction of ISGs, ISG15 and IFITM1 by IFNα-2a is dependent on Akt/mTOR signaling and autophagy, as examined in the present study.

Type I IFN (IFN-I) can modulate JAK-STAT and PI3K/Akt/mTOR pathways, which induce autophagy and drive downstream biological activities. IFNα induces rapid tyrosine phosphorylation of the insulin receptor substrate-1 (IRS-1) and then activates PI3’-kinase was firstly demonstrated in U-266 and in Daudi cells (Uddin et al., 1995). Lekmine et al. (2004) found that type I IFN activated the proteins downstream of PI3K pathway including p70 S6K and 4E-BP1. Schmeisser et al. confirmed that type I IFN induces autophagy and particularly, the autophagosomes were induced by type I IFNs in Daudi and T98G cells as shown by electron microscopy (Schmeisser et al., 2013). IFNs induces autophagy through IGF1–PI3K/Akt/mTOR signaling pathways and participates in controlling tumor growth, inflammatory reactions and antiviral activities (Degtyarev et al., 2008; Gu et al., 2017; Perot et al., 2018; Ma et al., 2019). Leukocyte IFN failed to increase autophagosomes formation after JAK1 or STAT1 knockout, which highlighted the role of JAK/STAT pathway in IFN-α-induced autophagy in antitumor activity (Zhu et al., 2013). Therefore, IFNα triggers autophagy *via* both canonical and non-canonical signaling pathways and may contribute to the outcome of IFNα treatment in different diseases. Our results verified that IFNα-2a induces autophagy by Akt/mTOR in hepatic cells.

In our study, as shown in **Figures 1** and **3**, IFNα-2a treatment decreased the phosphorylation of Akt/mTOR and increased the autophagosome formation in hepatocytes. IFNα-2a downregulated the phosphorated form of Akt, and it cannot be recovered by insulin-mediated Akt activation, suggesting that IFNα-2a may also interfere with some other upstream proteins expression and functions.

Additionally, cellular autophagy process can be modulated by different glucose concentrations through AMPK signaling pathways (Wang et al., 2020). Low cellular energy metabolism activates AMPK and initiates autophagy. In our study, even though IFNα-2a treatment downregulated the phosphorylated form of AMPK, it did not interfere with LC3II expression. In addition, using AICAR to upregulate the activity of AMPK increased the expression of LC3II in IFNα-2a and AICAR-cotreated cells. Thus, IFNα-2a may also significantly disturb AMPK phosphorylation in HepG2.2.15 cells. Besides, blocking autophagic degradation by suppressing lysosomal acidification can promote the expression of LC3 (Lin et al., 2020). In this study, the expression of p62, the cargo for autophagic degradation, was increased in IFNα-2a-treated cells. Consistently, the DQ-BSA assay indicated reduced autophagic degradation after IFNα-2a treatment, leading to the accumulation of autophagosomes.

However, the situation in PHHs was completely different. Since PHHs are not growing cells when cultured in the medium, thus with very low basal levels of phosphorylated mTOR and Akt. The levels of phosphorylated Akt and mTOR increased rapidly in response to stimulation of different doses of IFNα-2a, indicating that IFN activated the non-canonical pathways. Upon HBV infection, the levels of phosphorylated Akt and mTOR are increased accompanied with IFNα-2a treatment. Several studies indicated that HBV infection leads to the accumulation of HBsAg in the endoplasmic reticulum and thereby activates the PI3K/Akt/mTOR pathway (Yang et al., 2009). We previously reviewed that mTOR pathway is a central regulator of cell growth, metabolism, proliferation, survival and autophagy and how this pathway is regulated in HBV infection (Wang et al., 2021). AMPK is a sensitive indicator of the cytosolic AMP/ATP ratio (Towler and Hardie, 2007). Treatment with interferons may cause a reduction in cellular ATP levels (Lewis et al., 1996), thus, IFN-α 2a likely induces an increased level of phosphorylated AMPK indirectly through changes in the AMP/ATP ratio in PHHs. Our previous study revealed AMPK positively regulates autophagy and thereby increased HBV replication in PHHs (Wang et al., 2020), which is consistent with this finding in IFNα-2a treated PHHs. Overall, naïve or transiently infected PHHs differently respond to IFNα treatment than persistently infected cells with high levels of HBV replication and gene expression.

Previously, the antiviral activity against IFNα-2a was tested in different cell systems (Hayashi and Koike, 1989; Caselmann et al., 1992; Rang et al., 2001; Shen et al., 2018; Chen et al., 2021). IFNα subtype 14 showed a higher potency to reduce HBV replication by simultaneously eliciting IFN-α and -γ signaling in PHHs. Thus, type I IFNs are able to modulate other cellular signaling pathways and thereby exert antiviral activities.

A relatively high dose of IFNα-2a was required to suppress HBV replication in the primary human hepatocytes (PHHs) (Chen et al., 2021). In hepatoma cells, HBV suppression could be achieved if the cells were treated with IFNα-2a prior to or early after transfection with replication-competent HBV genomes (Rang et al., 1999). This is reproducible in our own experiments (data not shown). Moreover, suppression of established HBV replication in hepatic cells by IFNα-2a is rather ineffective (Hayashi and Koike, 1989; Caselmann et al., 1992). In our cell culture system, HBV replication was slightly enhanced when treated with 1000 or 6000 U/ml of IFNα-2a. We also attempted to achieve stronger antiviral activities by increasing IFNα-2a concentrations up to 24000 U/ml to HepG2.2.15 cells. The high doses of IFNα-2a increased ISGs expression but did not reduce HBV replication and gene expression in HepG2.2.15 cells (**Figure S4**). Thus, it is not likely to achieve stronger HBV suppression by escalating doses of IFNα-2a in this cell system. It is consistent with Hayashi et al. (Hayashi and Koike, 1989) and Caselmann’s (Caselmann et al., 1992) studies that HBV replication and HBsAg production were not changed after treatment with IFNs in HBV-producing cell lines. Thus, IFNα-2a may preferentially protect uninfected hepatocytes against the establishment of HBV infection while it does not effectively clear HBV from persistently infected cells. Recently, Wu et al. investigated the hepatic gene expression profiles in patients received IFNα treatment (Wu et al., 2016). They found that non-responder patients have elevated ISGs expression prior to the treatment. It is generally assumed that HBV clearance is not achieved by the direct antiviral activity of IFNs rather by their ability for immune modulation (Pei et al., 2014).

Further, the interaction of HBV and IFN signaling is also very complex. A recent study showed that HBV may escape from the host innate immune system by promoting a complex composed of HK2, MAVS and VDAC1, which required Akt activity (Zhou et al., 2021). Besides, Li et al. (2021) reported that IFN-α inhibited the expression of MAVS in HepG2.2.15, thereby weakening its anti-HBV effect.

IFNs can activate a signal transduction cascade and induce the expression of ISGs. In the present study, it was demonstrated that the expression of ISGs, ISG15 and IFITM1, was attenuated by blocking autophagy. These results indicate that induction of ISG15 and IFITM1 is dependent on the initiation of autophagy. However, IFNα-2a-induced ISG15 and IFITM1 expressions are differently regulated at low and high glucose conditions. The activation of the Akt/mTOR pathway may also allow ISG15 expression (Kaur et al., 2008; Kaur et al., 2012). Our results revealed that the high glucose concentration modulated higher expression levels of Akt and mTOR, which may contribute to the expression of ISG15.

In conclusion, our findings demonstrated that IFNα-2a can inhibit Akt/mTOR signaling pathway, resulting in the initiation of autophagy and blockade of autophagic degradation. This non-canonical IFNα signaling may positively modulate HBV replication in hepatic cells.

## Data Availability Statement

The raw data supporting the conclusions of this article will be made available by the authors, without undue reservation.

## Author Contributions

ML contributed to the conception of this study. JL performed the experiment and data analyses and wrote the manuscript. TK offered excellent technical support. RB provided PHHs. JC and ZY helped perform the analysis with constructive discussions. XW aided in interpreting the results and worked on the manuscript. All authors contributed to the article and approved the submitted version.

## Funding

This work was supported by a grant from the Deutsche Forschungsgemeinschaft (RTG1949) and a scholarship from the Medical Faculty of University Duisburg-Essen.

## Conflict of Interest

The authors declare that the research was conducted in the absence of any commercial or financial relationships that could be construed as a potential conflict of interest.

## Publisher’s Note

All claims expressed in this article are solely those of the authors and do not necessarily represent those of their affiliated organizations, or those of the publisher, the editors and the reviewers. Any product that may be evaluated in this article, or claim that may be made by its manufacturer, is not guaranteed or endorsed by the publisher.

## Abbreviations

HBV, Hepatitis B virus; ISGs, IFN-stimulated genes; ISG15, Interferon Stimulated Gene 15; IFITM, interferon-induced transmembrane proteins; LC3, microtubule‐associated protein 1 light chain 3 beta.
