# Supplementary material for: Interferon Alpha Induces Cellular Autophagy and Modulates Hepatitis B Virus Replication
Source: Front Cell Infect Microbiol. 2022 Feb 2;12:804011. doi: 10.3389/fcimb.2022.804011 (PMC8847603; doi:10.3389/fcimb.2022.804011)
Supplement: Supplementary file 1 [file DataSheet_1.docx]

**Supplement Figures**

**Figure S1**

**
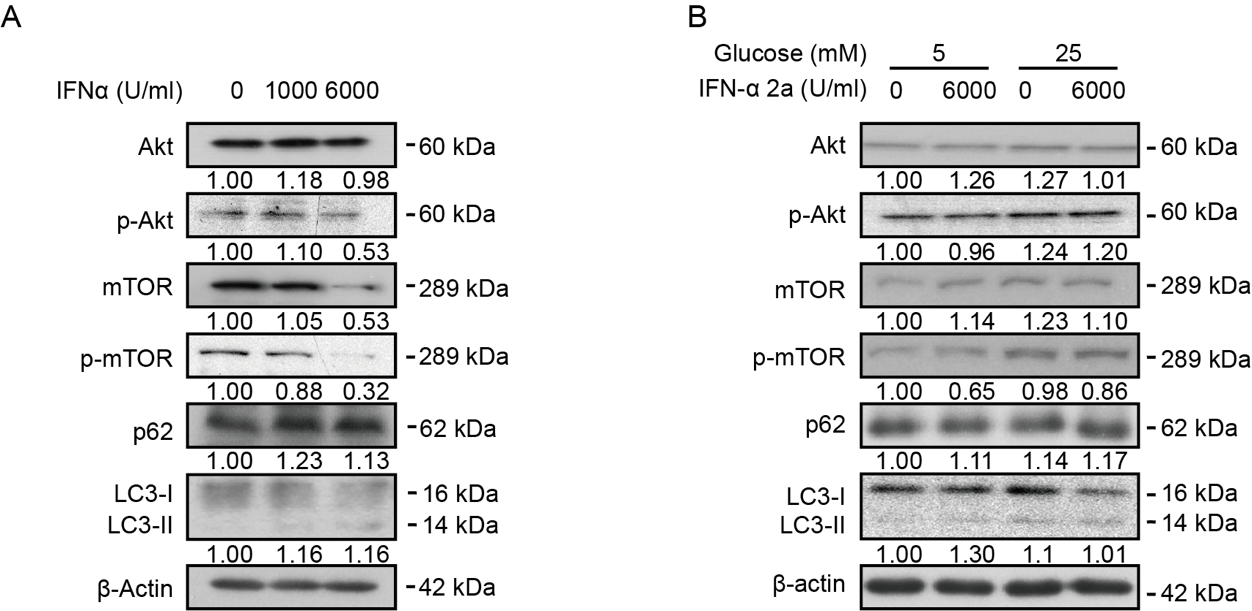
**

***Figure S1.*** ***IFNα-2a inhibits Akt/mTOR activation and enhances autophagy independently on glucose concentrations in Huh7 cells***

**(A)** Huh7 cells were incubated in medium and treated with indicated concentrations of IFNα-2a (1000 U/ml and 6000 U/ml) for 48 hr. **(B)** Huh7 cells were cultured in medium with the indicated glucose concentrations (5 and 25 mM) with or without 6000 U/ml IFNα-2a and harvested after 48 hr. The total and phosphorylated Akt, mTOR, and p62 and LC3 were detected by western blotting and their relative levels were determined by quantifying the gray scales of bands, using beta‐actin as a loading control.

**Figure S2**


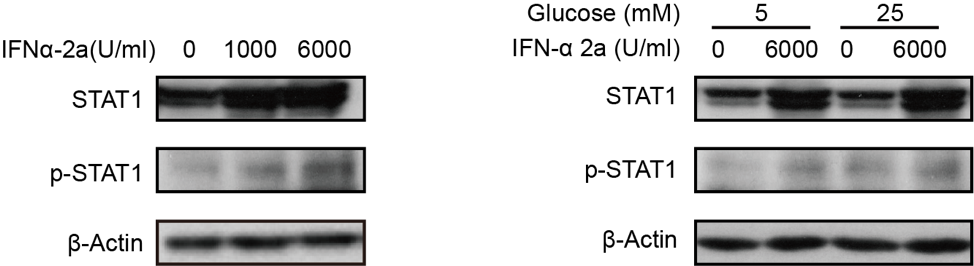


***Figure S2. IFN-α activates STAT signaling pathway in HepG2.2.15 cells.***

HepG2.2.15 cells were treated with the indicated concentrations of IFNα-2a and with or without the indicated glucose concentrations (5 and 25 mM) and harvested after 48 hr. The total and phosphorylated STAT1 were detected by western blotting and their relative levels were determined by quantifying the gray scales of bands, using beta‐actin as a loading control.

**Figure S3**

**
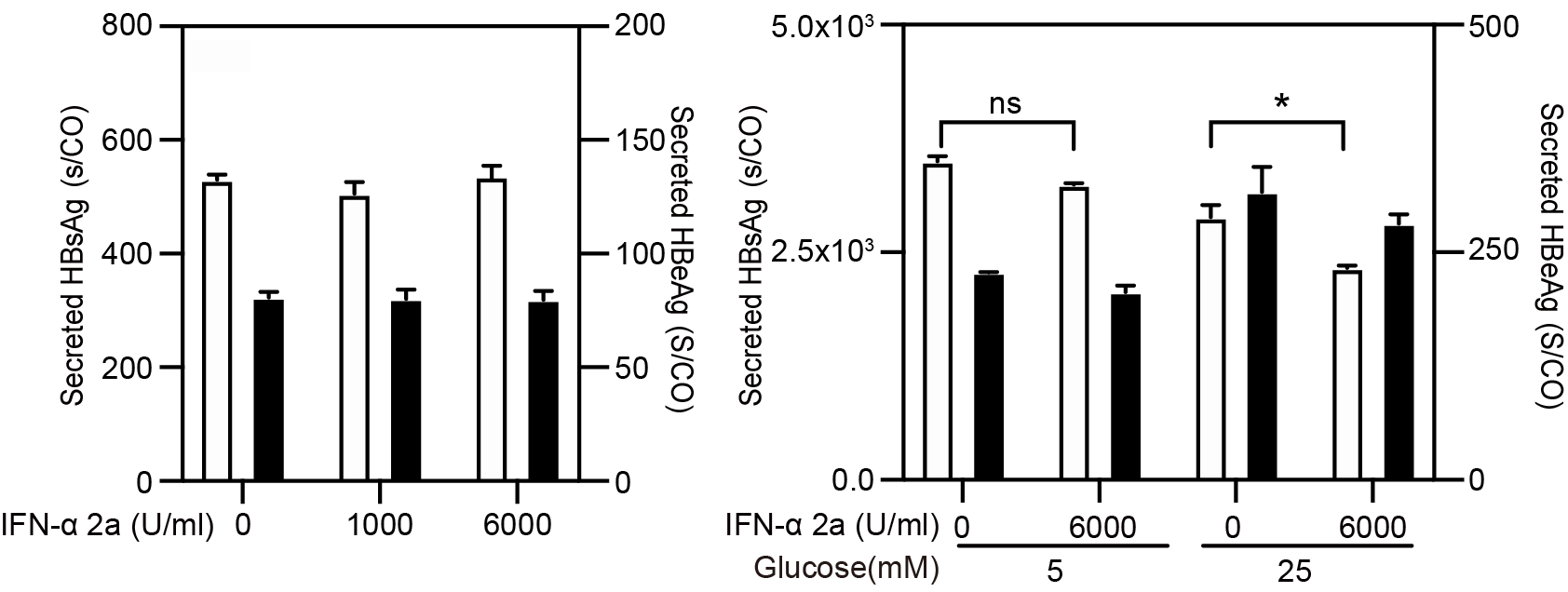
**

***Figure S3.*** ***High IFNα-2a concentrations do not inhibit HBV replication and gene expression in Hepatoma cells.***

Huh7 cells were cultured in medium and treated with the indicated IFNα-2a (1000 U/ml and 6000 U/ml). Huh7 cells were cultured in medium and treated with the indicated glucose concentrations (5 and 25 mM) with or without 6000 U/ml IFNα-2a and harvested after 48 hr. After 72 hr, the HBsAg and HBeAg levels in the culture supernatants were quantified by chemiluminescent microparticle immunoassay (CMIA). ns, not significant; *P < 0.05.

**Figure S4**


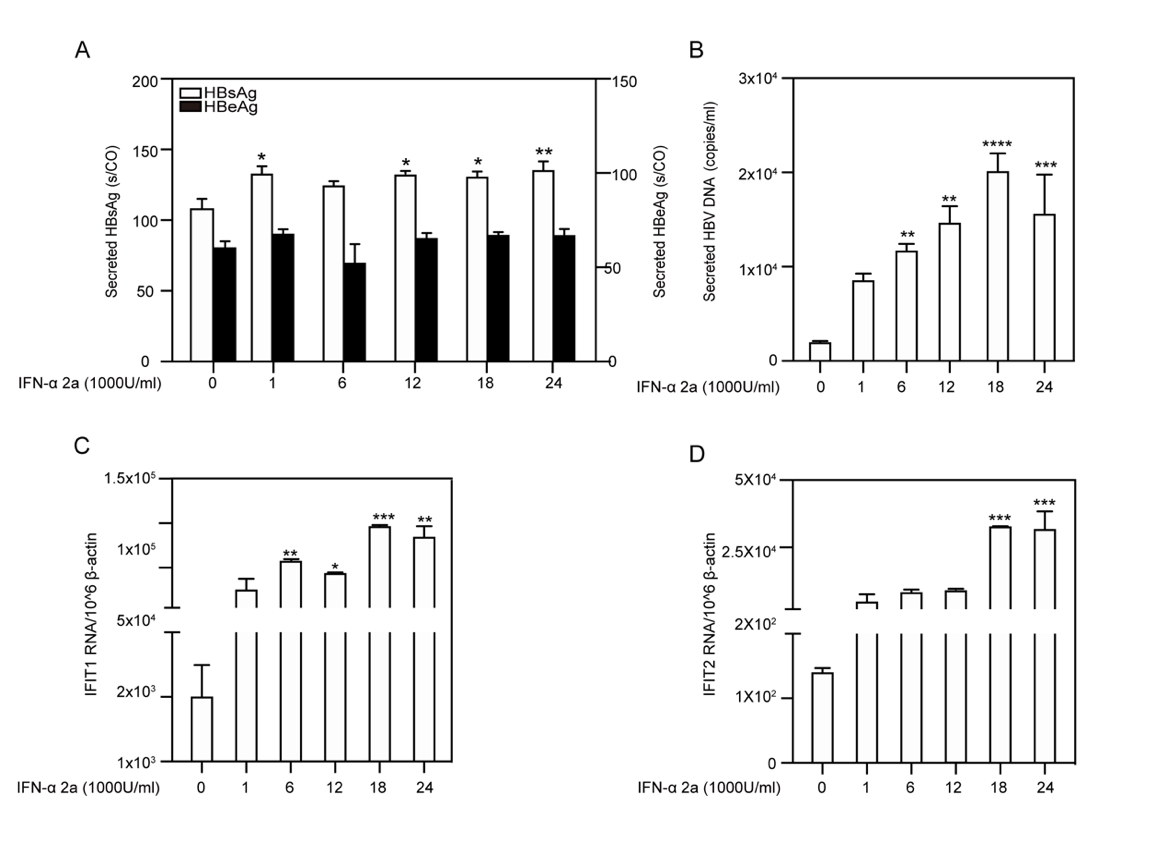


***Fig S4. The high doses of IFNα-2a increased ISG expression but did not reduce HBV replication in HepG2.2.15 cells.***

**(A-D)** HepG2.2.15 cells were cultured in medium with the indicated concentrations of IFNα-2a. After 72 hr, cells were harvested. **(A)** The HBsAg and HBeAg levels in the culture supernatants and intracellular HBsAg and HBeAg from cell lysates were quantified by chemiluminescent microparticle immunoassay (CMIA). **(B)** The total HBV DNA levels in the supernatants were measured by real-time PCR. **(C)** Real‐time RT‐PCR was performed to determine the IFIT1 and IFIT2 RNA levels in HepG2.2.15 cells. *p < .05; **p < .01; ***p < .001; ****p < .0001.

**Figure S5**


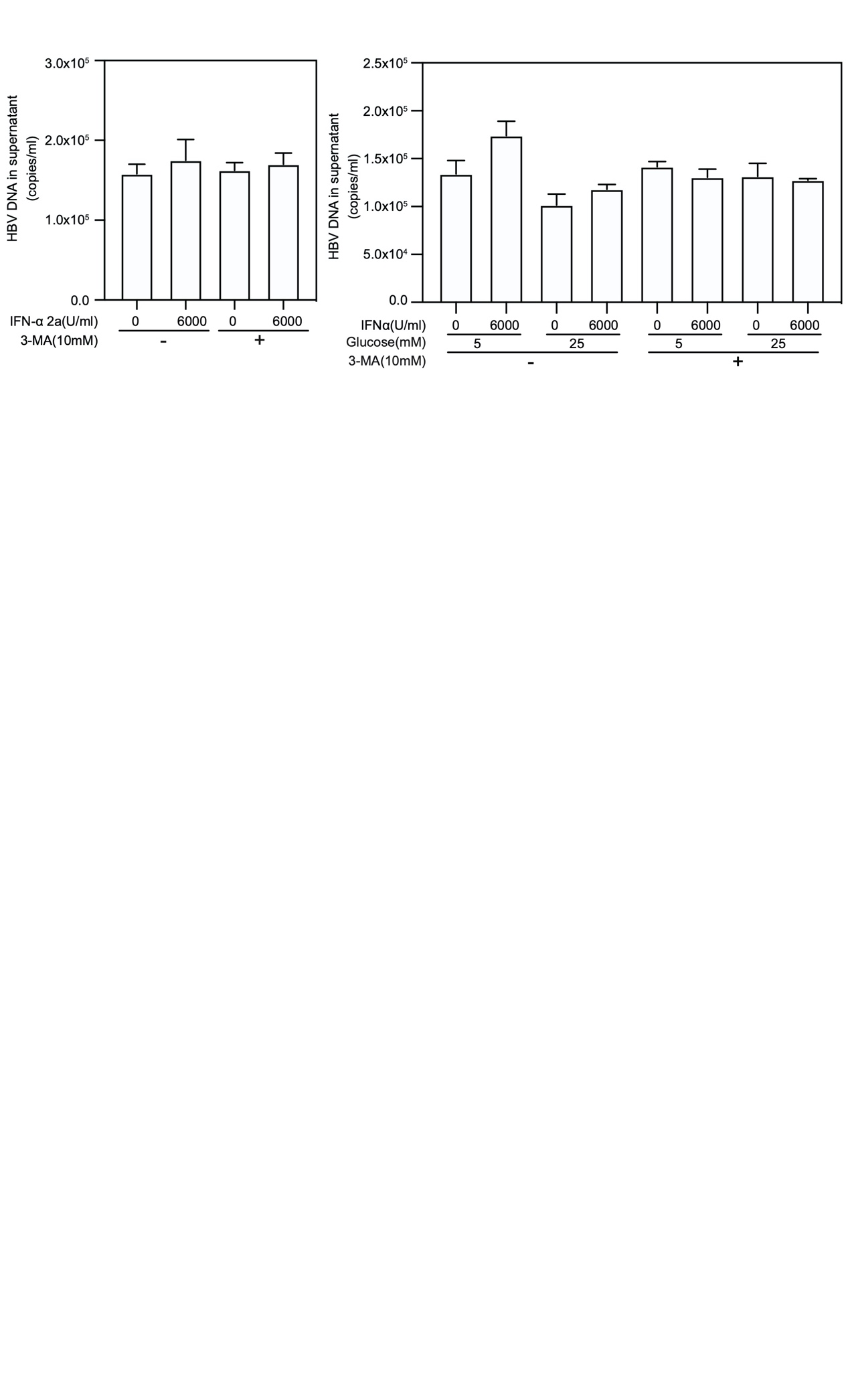


***Figure S5. The treatment of IFNα-2a doesn’t inhibit the production of HBV progeny.***

HepG2.2.15 cells were treated with 6000 U/ml IFNα-2a with or without 10 nM 3-MA. Or HepG2.2.15 cells were cultured at the indicated glucose concentrations (5 mM and 25 mM) and 6000 U/ml IFNα-2a with or without 10 nM 3-MA. After 72 hr, the levels of HBV DNA in the supernatants were determined by real-time PCR.
